# A natural genetic variation screen identifies insulin signaling, neuronal communication, and innate immunity as modifiers of hyperglycemia in the absence of *Sirt1*

**DOI:** 10.1093/g3journal/jkac090

**Published:** 2022-04-18

**Authors:** Rebecca A S Palu, Katie G Owings, John G Garces, Audrey Nicol

**Affiliations:** 1 Department of Biological Sciences, Purdue University-Fort Wayne, Fort Wayne, IN 46818, USA; 2 Department of Human Genetics, University of Utah School of Medicine, Salt Lake City, UT 84112, USA

**Keywords:** hyperglycemia, *Drosophila*, genetic variation, modifier genes

## Abstract

Variation in the onset, progression, and severity of symptoms associated with metabolic disorders such as diabetes impairs the diagnosis and treatment of at-risk patients. Diabetes symptoms, and patient variation in these symptoms, are attributed to a combination of genetic and environmental factors, but identifying the genes and pathways that modify diabetes in humans has proven difficult. A greater understanding of genetic modifiers and the ways in which they interact with metabolic pathways could improve the ability to predict a patient’s risk for severe symptoms, as well as enhance the development of individualized therapeutic approaches. In this study, we use the *Drosophila* Genetic Reference Panel to identify genetic variation influencing hyperglycemia associated with loss of *Sirt1* function. Through analysis of individual candidate functions, physical interaction networks, and gene set enrichment analysis, we identify not only modifiers involved in canonical glucose metabolism and insulin signaling, but also genes important for neuronal signaling and the innate immune response. Furthermore, reducing the expression of several of these candidates suppressed hyperglycemia, making them potential candidate therapeutic targets. These analyses showcase the diverse processes contributing to glucose homeostasis and open up several avenues of future investigation.

## Introduction

Metabolic diseases, and in particular diabetes, are one of the most pressing health crises in the developed world, with incidences continuing to rise in the last 20 years (CDC 2020). A total of 37.7% of adults in the United States are diagnosed as obese and 10.5% as having some form of diabetes, and it is estimated that millions more go undiagnosed (Flegal *et al.* 2016; CDC 2020). What is more, the monetary cost of these disorders to the public has grown to astronomical levels. It is estimated that $237 billion in direct costs and at least $90 billion in indirect costs were spent on healthcare related to diabetes and its various complications in 2017 alone, up ∼25% from 2012 (Flegal *et al.* 2016). A focused effort has been made to understand both the genetic and environmental contributors to metabolic homeostasis, as well as to the disruption of that homeostasis that leads to disease (Barroso and McCarthy 2019).

Unfortunately, even identifying these contributors has proven difficult. Metabolic diseases are complex, and the onset, progression, and ultimately the severity of any individual case is dependent upon a myriad of genetic and environmental variables and the ways in which they interact with one another (Queitsch *et al.* 2012; Barroso and McCarthy 2019). Even when there is a strong familial link, phenotypic heterogeneity in disease phenotypes can make it difficult to identify at-risk patients or make accurate prognostic predictions (Udler *et al.* 2019). This is particularly true when it comes to predicting complications of diabetes such as neuropathy, retinopathy, or kidney disease (Barroso and McCarthy 2019; Cabrera *et al.* 2020). Much of this variation is due to interindividual differences in genetic background, including silent cryptic genetic variation that is revealed upon disease or stress (Queitsch *et al.* 2012; Chow 2016; Barroso and McCarthy 2019).

One example of this kind of symptom heterogeneity can be observed in disease associated with the deacetylase *SIRT1*. This highly conserved gene was originally identified as a histone deacetylase important in heterochromatin formation in yeast (Shore *et al.* 1984; Ivy *et al.* 1986; Rine and Herskowitz 1987). Since then, *SIRT1* and its paralogs (the sirtuins) have been found to have a number of additional targets, many of which are transcription factors and enzymes with key roles in metabolic homeostasis (Brunet *et al.* 2004; Picard *et al.* 2004; Li *et al.* 2007; Rodgers and Puigserver 2007; Yang *et al.* 2009; Palu and Thummel 2016). Importantly, as part of their enzymatic reaction, sirtuins consume the cofactor NAD, which also serves as an electron carrier in central metabolic pathways such as glycolysis and the TCA cycle. Sirtuin enzymatic activity, therefore, is directly linked to the availability of this cofactor and thus is responsive to the energetic state of the cell. This information is then conveyed to the targets, whose acetylation state alters their activity and stability in the cell (Nogueiras *et al.* 2012). With this centralized role in regulating the response of metabolic factors to cellular energy availability, it is unsurprising that variation in *SIRT1* has been linked to, among other things, the development of diabetes (Zillikens *et al.* 2009; Botden *et al.* 2012; Biason-Lauber *et al.* 2013; Zhao *et al.* 2017).

Elucidating the mechanism behind this link, however, has proven difficult. Loss-of-function and gain-of-function studies in model systems have demonstrated a clear role for *SIRT1* in metabolic homeostasis, but the actual impacts on the animals in question have frequently been contradictory (Boutant and Cantó 2014). For example, overexpression of *SIRT1* in the liver has in separate studies been linked to both increased and decreased glucose production (Rodgers and Puigserver 2007; Wang *et al.* 2010). Complete loss of *SIRT1* in mice has led to phenotypes ranging from embryonic lethality to survival to adulthood with metabolic dysfunction depending on the strain (McBurney *et al.* 2003; Boily *et al.* 2008). It is likely that at least some of these contradictory results stem from differences in genetic background between the animals used in the various studies. Understanding the role of this variation and the genes or pathways which modify metabolic disease will enable the development of improved diagnosis, prediction of prognosis, and personalized treatment strategies for patients.

Model organism tools, such as the *Drosophila* Genetic Reference Panel (DGRP), provide a way to study of the impact of natural genetic variation on diseases such as diabetes (Mackay *et al.* 2012; He *et al.* 2014; Ivanov *et al.* 2015; Nelson *et al.* 2016; Jehrke *et al.* 2018; Everman *et al.* 2019). The DGRP is a collection of ∼200 isogenic strains derived from a wild population, such that each strain represents one wild-derived genome (Mackay *et al.* 2012). The variation in the DGRP is well tolerated under healthy, nondisease conditions and allows for the identification of genetic polymorphisms that are associated with phenotypic variation in models of human disease (Chow and Reiter 2017). Importantly, the availability of full-genome sequence for these strains allows for genome-wide association (GWA) analyses that link quantitative phenotypes with genetic variation and modifier genes.

The utility of the DGRP in identifying candidate modifiers of metabolic disease has already been demonstrated numerous times, with screens associated with misfolded insulin, high sugar and high fat feeding, and starvation resistance already documented (Mackay *et al.* 2012; He *et al.* 2014; Ivanov *et al.* 2015; Nelson *et al.* 2016; Jehrke *et al.* 2018; Everman *et al.* 2019). While some of these used biochemical assays to precisely measure metabolite levels in flies as a quantitative phenotype for the screen (Nelson *et al.* 2016; Jehrke *et al.* 2018; Everman *et al.* 2019), several used more general physiological measurements such as starvation resistance and lifespan (Mackay *et al.* 2012; Ivanov *et al.* 2015). Although effective, the use of this kind of general readout reduces the specificity of the modifiers identified. Many genetic factors impact survival and could lead to a high background signal. The same could be true for otherwise wild-type flies subjected to different environmental conditions, even when more precise assays are used as a quantitative phenotype (Nelson *et al.* 2016). A multitude of pathways and processes impact the response to dietary changes through feeding rate, hormone secretion, anabolism and catabolism rates, and nutrient absorption. Using a specific genetic model of disease and then focusing on a specific phenotype, such as hyperglycemia, may reduce some of the noise and increase specificity of the modifiers identified.

Furthermore, recent studies using the DGRP have demonstrated that the top candidate modifier genes and pathways differ when different but related models of genetic disease are screened (Chow *et al.* 2016; Palu *et al.* 2019). These results reinforce the idea that different causative genes and mutations will interact with different pathways over the course of disease. It also highlights the importance of exploring multiple disease models in something so diverse as diabetes, and the utility of the DGRP in precisely distinguishing modifiers of a particular genotype-phenotype combination.

In this study, we report the results of a natural variation screen in a model of *Sirt1* loss of function. Loss of this gene in *Drosophila* has been shown to lead to progressive metabolic dysfunction including obesity, hyperglycemia, and ultimately insulin resistance (Palu and Thummel 2016). Our study design is specifically focused on the phenotype of hyperglycemia when *Sirt1* expression is disrupted using RNAi in the adipose and liver-like fat body organ (Géminard *et al.* 2009; DiAngelo and Birnbaum 2009; Arrese and Soulages 2010). While there are likely roles for *Sirt1* in other metabolic tissues, its function in the fat body clearly contributes to the maintenance of glucose homeostasis and insulin sensitivity over time (Palu and Thummel 2016). We observed substantial phenotypic variation across the DGRP for hyperglycemia associated with loss of *Sirt1*. Using GWA analysis, pathway enrichment, and the generation of a physical interaction network, we identified a number of modifying pathways and processes, several of which have known roles in central carbon metabolism, the immune response, and the kind of neuronal signaling and communication expected to influence the neuroendocrine cells responsible for insulin secretion in *Drosophila*. Finally, we confirmed that reduction in the expression of several of the top candidate modifier genes significantly alters glucose levels in the *Sirt1* RNAi model. Our findings highlight exciting new areas of study for modifiers of *Sirt1* function, glucose homeostasis, and insulin sensitivity.

## Materials and methods

### Fly stocks and maintenance

Flies were raised at room temperature on a diet based on the Bloomington *Drosophila* Stock Center standard medium with malt. Experimental crosses were maintained on a media containing 6% yeast, 6% dextrose, 3% sucrose, and 1% agar, with 0.6% propionic acid and 0.1% *p*-hydroxy-benzoic acid methyl ester in 95% ethanol included as antifungal agents. Flies subjected to an overnight fast were transferred to media containing only 1% agar in water. The *r4>Sirt1-RNAi* strain, which serves as the model of hyperglycemia in this study, is derived from an *r4-GAL4* strain (BDSC 33832) outcrossed to *w^1118^* (Palu and Thummel 2016) and a *Sirt1* RNAi strain (32481) from the Bloomington *Drosophila* Stock Center (*w−/w−; +/+; [r4-GAL4, UAS-Sirt1-RNAi]/[r4-GAL4, UAS-Sirt1-RNAi]*). 185 strains from the DGRP were used for the modifier screen (Supplementary Tables 1–3), wherein virgin females carrying the model were crossed to males of the DGRP strains. Male F1 progeny carrying *r4>Sirt1-RNAi* were separated and aged for 1–2 weeks. These flies were then either collected under ad libitum fed conditions or fasted overnight and then collected. The following RNAi and control strains are from the Bloomington *Drosophila* Stock Center: *CG4168* RNAi (28636), *CG5888* RNAi (62175), *uif* RNAi (38354), *CTPSyn* RNAi (31924), *smt3* RNAi (36125), *ilp5* RNAi (33683), *Vha55* RNAi (40884), *snRNP-U1-70k* RNAi (33396), *CG10265* RNAi (43294), *CG15803* RNAi (51449), *Roe* RNAi (57836), *CG34353* RNAi (58291), *CadN2* RNAi (27508), *Ace* RNAi (25958), *CG43897* (31560), *dsxc73A* RNAi (56987), *bgm* RNAi (56979), *CG3407* RNAi (57762), control *attP40* (36304), and control *attP2* (36303). Additional RNAi lines were also obtained and tested from the Bloomington *Drosophila* Stock Center for *bgm* (55918, 28639), *CTPSyn* (31752, 53378), *dsxc-73A* (56964), *ilp5* (31378), *smt3* (28034), and *uif* (38365) (data not shown).

### Glucose assay

Glucose was measured specifically in males. Adult female *Drosophila* devote much of their physiological output to egg production (Millington and Rideout 2018). The consequence of this is that metabolic changes are frequently buffered or undetectable in nonvirgin females. In contrast, adult males have proven to be a consistent model for metabolic homeostasis in the fly while also being easier to collect and maintain than virgin females. When that homeostasis is disrupted, physiological changes are readily detectable in males (Sieber and Thummel 2009; Tennessen *et al.* 2014; Barry and Thummel 2016; Palu and Thummel 2016; Beebe *et al.* 2020).

Samples of 5 flies each were collected at 1 or 2 weeks of age and washed in 1XPBS. Samples were then either frozen in liquid nitrogen and stored long term at −80°C or immediately homogenized in 100 µl 1× PBS. Samples were kept frozen until immediately upon addition of PBS and homogenization. After homogenization samples were subjected to heat inactivation of enzymes at 70°C for approximately 10 min. Lysates could then be stored long term at −80°C. Samples were centrifuged at ∼16,000 × g for up to 5 min at room temperature and glucose was measured undiluted from the supernatent using the Sigma HK Glucose Assay kit as described (Tennessen *et al.* 2014). This is a quantitative assay where higher concentrations of glucose correlate with more severe hyperglycemia, and lower concentrations of glucose correlate with milder disease, or potentially hypoglycemia.

### Protein assay

Prior to heat inactivation, 10 µl of the fly lysate isolated for glucose measurement was saved and kept on ice. Protein samples could then be stored long term at −80°C. Samples were centrifuged at ∼16,000 × g for up to 5 min at room temperature, and protein was measured from the supernatant after a 1:10 dilution using the Sigma Protein Assay Reagent as described (Tennessen *et al.* 2014).

### Phenotypic analysis and genome-wide association

For each DGRP line, glucose was measured from 3 samples of 5 flies each aged to 13–16 days posteclosion and fasted for 12–13.5 h. The *P*-values for association of genetic background and glucose concentration were calculated using 1-way ANOVA on R software taking into account all collected data points for each experiment. Average glucose concentration was used for the GWA. GWA was performed as previously described (Chow *et al.* 2016; Palu *et al.* 2019). DGRP genotypes were downloaded from the website, http://dgrp.gnets.ncsu.edu/. Nonbiallelic sites were removed. A total of 3,636,891 variants were included in the analysis. Mean eye glucose concentration for 555 samples representing 2775 DGRP/*r4>Sirt1-RNAi* F1 progeny were regressed on each SNP. To account for cryptic relatedness (He *et al.* 2014; Huang *et al.* 2014), GEMMA (v. 0.94) (Zhou and Stephens 2012) was used to both estimate a centered genetic relatedness matrix and perform association tests using the following linear mixed model (LMM):

y=α+xβ+u +∈

u ∼ MVN_n(0,λT^(-1)K)

∈∼MVN_n(0,T^(-1)1_n)

where, as described and adapted from Zhou and Stephens (2012), *y* is the *n*-vector of average glucose concentration for the n lines, α is the intercept, *x* is the *n*-vector of marker genotypes, β is the effect size of the marker. *u* is a *n* × *n* matrix of random effects with a multivariate normal distribution (MVN_n) that depends on λ, the ratio between the 2 variance components, T^(−1), the variance of residuals errors, and where the covariance matrix is informed by K, the calculated *n* × *n* marker-based relatedness matrix. K accounts for all pairwise nonrandom sharing of genetic material among lines. ϵ, is a *n*-vector of residual errors, with a multivariate normal distribution that depends on T^(−1) and I_n, the identity matrix. Quantile-quantile plots demonstrate an appropriate fit to the LMM at the positive end of the plot, but a greater number of points than expected by chance with an insignificant *P*-value (Supplementary Fig. 1). Genes were identified from SNP coordinates using the BDGP R54/dm3 genome build. A SNP was assigned to a gene if it was ±1 kb from a gene body.

### RNAi validation

Virgin females from the *r4>Sirt1-RNAi* model were crossed to males carrying RNAi constructs targeting candidate modifiers of those models, and the glucose levels of F1 male progeny expressing both *Sirt1i* and the modifier RNAi construct specifically in the fat body under the control of the *r4-GAL4* driver were measured as described above on 4–5 samples of 5 male flies each. Glucose concentrations from RNAi-carrying strains are compared directly to genetically matched *attP40* or *attP2* controls using a Dunnett’s multiple comparisons test. Glucose measurements are normalized to the appropriate genetically matched controls. Normalized controls from individual experiments are compared in Supplementary Fig. 2. Standard deviation did not significantly vary between controls for individual experiments.

### RNA isolation and qPCR

RNA was isolated from whole adult male flies, or from adult abdominal fat body attached to cuticle using the NEB Monarch RNA Isolation Kit with DNAse digestion. cDNA was generated using the ThermoFisher Verso cDNA Synthesis Kit with Olido-dT primers. Expression of *Sirt1* and the 3 candidate suppressor modifiers (*CG4168*, *CG5888*, and *uif*) was analyzed using qPCR (Supplementary Table 1). Expression was normalized using *rpl19* expression as a control (Supplementary Table 1).

### Bioinformatics analysis

Genetic polymorphisms were associated with candidate genes within 1 kb of the polymorphism. Information about candidate genes and their human orthologs was gathered from a number of databases including Flymine, Flybase, OMIM, and NCBI, then verified through primary sources. Physical interaction maps were generated using the GeneMANIA plugin on Cytoscape (version 3.8.2) (Shannon *et al.* 2003; Montojo *et al.* 2010). Data on the GeneMANIA database is pulled from a variety of physical interaction networks including immunoprecipitation, yeast two-hybrid, and specific single-gene studies (Warde-Farley *et al.* 2010). GSEA was run to generate a rank-list of genes based on their enrichment for significantly associated polymorphisms. For GSEA analysis, polymorphisms within 1 kb of more than 1 gene were assigned to one gene based on a priority list of exon, UTR, intron, and upstream or downstream. Genes were assigned to GO categories, and calculation of enrichment score was performed as described (Subramanian *et al.* 2005). Categories with ES scores > 0 (enriched for associated genes with low *P*-values), gene number > 3, and *P*-values <0.05 were included in the final output.

## Results

### Glucose levels in *r4*>*Sirt1*-RNAi flies vary with genetic background in a consistent pattern across multiple conditions

Loss of *Sirt1* expression leads to progressive hyperglycemia, obesity, and insulin resistance. To model the hyperglycemia that is commonly associated with diabetes, we reduced the expression of the deacetylase *Sirt1* specifically in the fat body of *Drosophila melanogaster*. This is achieved using the *GAL4/UAS* system, with *r4-GAL4* driving expression of *UAS-Sirt1* RNAi (Supplementary Fig. 4a). *r4-GAL4* is strongly expressed primarily in the fat body of fly, starting in early development and continuing through adulthood (Lee and Park 2004). Sirt1 RNAi expressed in the fat body (*r4>Sirt1-RNAi*) reproduces the hyperglycemia phenotype, with an approximately 50–60% increase in whole fly glucose levels (*P* = 0.02, Supplementary Fig. 3) (Palu and Thummel 2016).

The line containing the model serves as the donor strain that was crossed to each DGRP strain. Females from the donor strain were crossed with males of each of 185 DGRP strains to generate F1 progeny lacking *Sirt1* expression in the fat body. The progeny received 50% of their autosomes from the maternal donor strain and 50% from the paternal DGRP strain (Supplementary Fig. 4b). Therefore, we are measuring the dominant effect of the DGRP background on the *Sirt1* RNAi hyperglycemia phenotype. This experimental design is similar to a model of *NGLY1* deficiency using RNAi that was also crossed to the DGRP (Talsness *et al.* 2020).

**Fig. 1.**
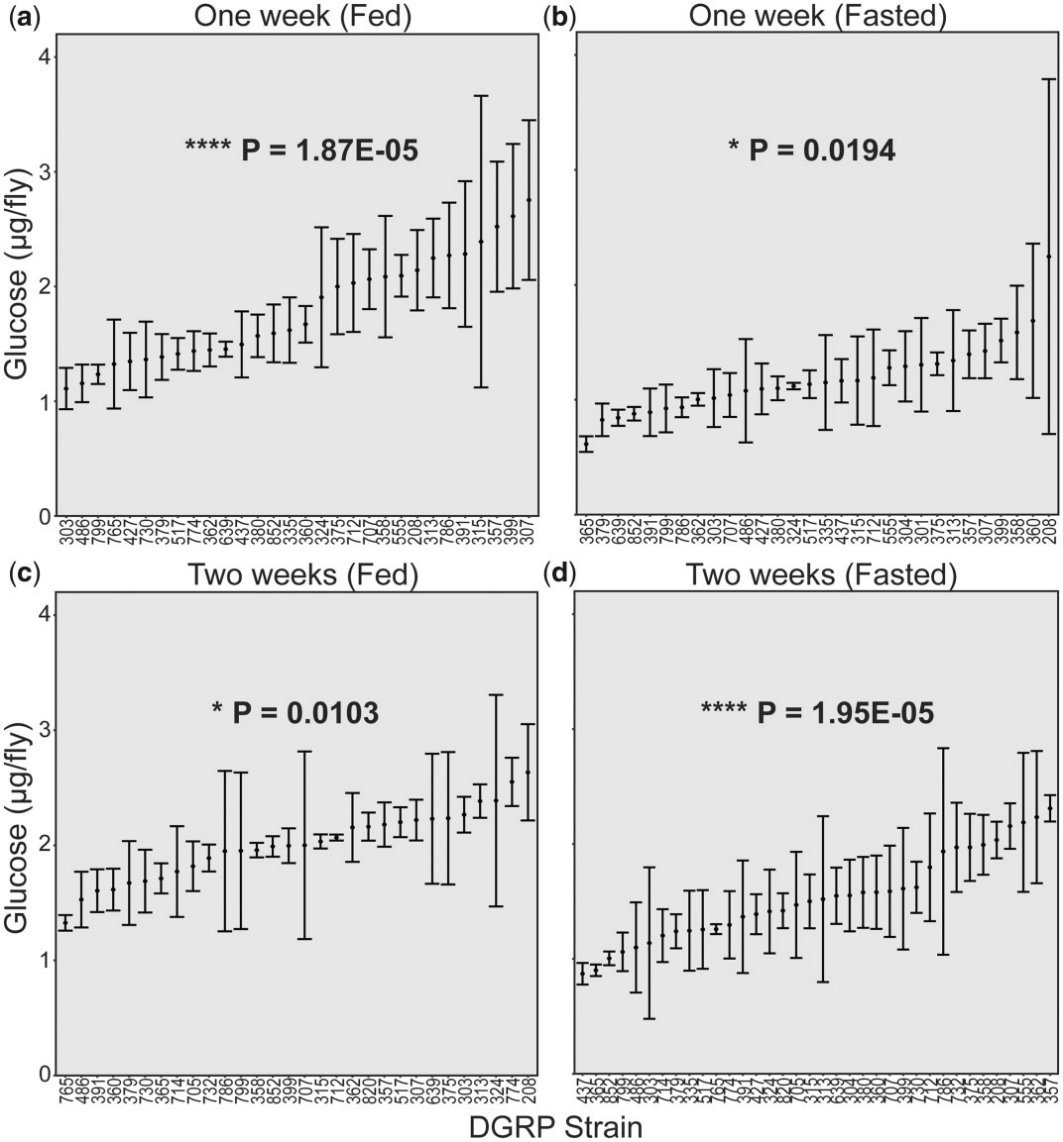
Glucose levels vary under a variety of environmental conditions. Glucose levels were measured in 3 samples for each of 30–36 strains under one of the indicated conditions: 1 week of adult age and fed ad libitum (*N* = 30 strains, *P* = 1.87E-05) (a), 1 week of adult age and fasted for 12–13 h (*N* = 30 strains, *P* = 0.0194) (b), 2 weeks of adult age and fed ad libitum (*N* = 30 strains, *P* = 0.0103) (c), and 2 weeks of adult age and fasted for 12–13 h (*N* = 36 strains, *P* = 1.95E-05) (d). Mean glucose concentrations are indicated, with error bars indicating standard deviation. DGRP strain or RAL numbers are indicated along the *X*-axis. *P*-values were calculated using 1-way ANOVA incorporating all individual measurements comparing DGRP strain with glucose concentration. Adult flies were collected within 2–3 days after eclosion from the pupal case and aged to the ages indicated at the top of the plot. *P < 0.05, ****P < 5E-05.

To ensure an appropriate set of conditions with respect to diet and age, we performed a preliminary analysis on 37 DGRP strains at 1 or 2 weeks of age, and under fasted or ad libitum fed conditions (Supplementary Table 2). Three samples were collected for glucose measurements in each strain and condition. Glucose levels vary across genetic background for each of the conditions being tested (Fig. 1, a–d). Average glucose for each strain is significantly correlated between 1 and 2 week fasted flies (*R* = 0.53, *P* = 4E-03), between 1-week-old fed and fasted flies (*R* = 0.45, *P* = 0.020), and between 1-week-old fed and 2-week-old fasted flies (*R* = 0.62, *P* = 2E-04) (Supplementary Fig. 5, a–c). This supports glucose concentration as a consistent quantitative measurement. Interestingly, evidence for correlation with 2-week-old flies fed ad libitum is not as strong. While a significant correlation is still detected with 1-week-old fasted flies (*R* = 0.43, *P* = 0.014), the correlation is not significant with 2-week-old fasted flies (*R* = 0.25, *P* = 0.219) and 1-week-old flies fed ad libitum (*R* = 0.34, *P* = 0.063) (Supplementary Fig. 5, d–f). By 2 weeks of age, *Sirt1* loss-of-function flies are beginning to experience more severe symptoms of disease, and we expect to see variability in symptoms and behavior in response to those symptoms. Fed flies at 2 weeks may have more variable glucose because feeding behavior is a big contributor to glucose levels in flies that have not been subjected to a fast.

To identify the conditions under which the impact of genetic background was the strongest, we performed a 1-way ANOVA test that included all data points collected. We found that while there is a significant association between glucose levels and genetic background under all conditions (*P* < 0.05), this effect is most pronounced in the 1-week-old flies fed ad libitum (*P* = 1.87E-5) and in the 2-week-old fasted flies (*P* = 1.95E-5) (Fig. 1, a and d). Because fasting reduces possible intrastrain variation caused by food in the gut, 2 weeks fasted was selected for the full screen.

We examined total protein concentration in 90 samples from the first 30 strains collected at 2 weeks fasted to ensure that any variation we observe in glucose is not due to differences in body size. Total protein levels do not significantly vary across the DGRP (*P* = 0.63, Supplementary Fig. 6a), nor do protein levels correlate with glucose levels in individual samples (*R* = 0.04, *P* = 0.6577, Supplementary Fig. 6b). We conclude that the variation we observe in fasting glucose levels are indeed due to differences in glucose and not to differences in body size.

### Genome-wide association analysis identifies candidate modifiers of *Sirt1i-*associated hyperglycemia

**Fig. 2.**
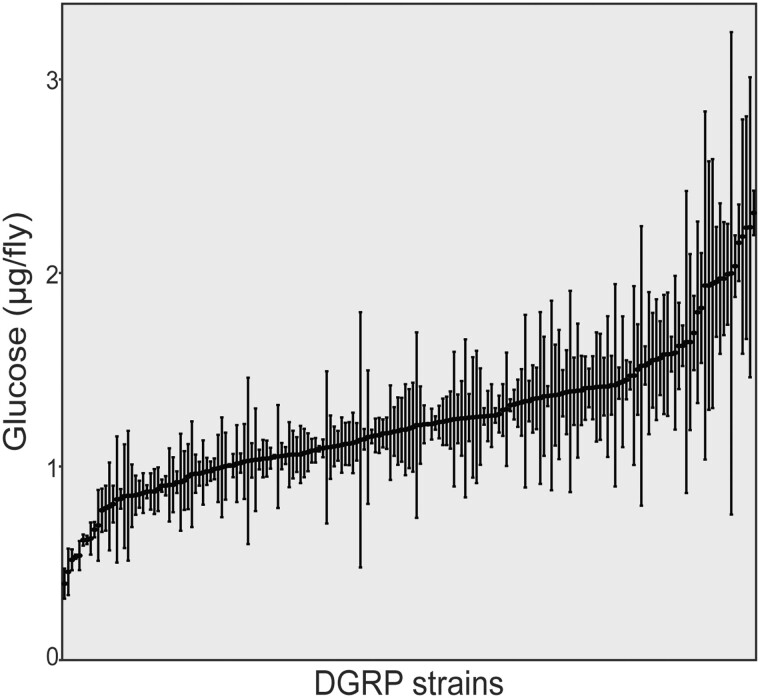
Glucose levels are significantly affected by genetic background. Glucose levels were measured in 3 samples for each of 185 strains at 2 weeks of age after a 12-h fast. Adult flies were collected within 2–3 days after eclosion from the pupal case and aged an additional 9–11 days prior to fasting (11–14 days posteclosion). Flies were collected after the overnight fast at 12–15 days posteclosion. Mean glucose concentrations are indicated, with error bars indicating standard deviation. DGRP strains along the *X*-axis are ordered from lowest to highest average glucose concentration. *P*-values were calculated using 1-way ANOVA incorporating all individual measurements comparing DGRP strain with glucose concentration (*P* < 2E-16).

Using the conditions determined in the preliminary screen, we proceeded to cross the donor strain with the remaining 149 DGRP strains (Fig. 2 and Supplementary Tables 2 and 3). We found a significant effect of genetic background on glycemia in the *r4>Sirt1-RNAi* flies (*P* < 2E-16) using 1-way ANOVA including all data points for each strain (*N* = 555). Individual glucose measurements ranged from 0.306  to 3.416 µg/fly (Supplementary Table 3), while average concentrations ranged from 0.395 µg/fly (RAL 801) to 2.438 µg/fly (RAL 357) (Fig. 2, Supplementary Table 4).

To identify genetic polymorphisms that may be responsible for this observed variation in glycemia, we performed a GWA analysis. Average glucose level for each strain was used as a quantitative phenotype to test for association with polymorphisms in the DGRP. A total of 3,636,891 variants were tested for the *r4>Sirt1-RNAi* model across 186 lines. This analysis as a result is insufficiently powered for candidates to remain statistically significant after multiple testing corrections. Instead, the focus is on identification of candidate modifiers and pathways that can be validated through further study and that will provide the basis for future projects. This approach has been quite successful in previous studies (Chow *et al.* 2013, 2015, 2016; Lavoy *et al.* 2018; Palu and Chow 2018; Palu *et al.* 2019, 2020; Talsness *et al.* 2020).

Because the analyzed F1 hybrids in this case were male and inherited their X chromosome from the donor strain and not the DGRP strain, we do not include any X-linked variants in the resulting candidate modifiers. Using an arbitrary cut-off of *P* < 10^−4^, we identified 237 polymorphisms on the second and third chromosomes (Supplementary Table 5). Of these 237, 62 were not considered further as they were not within ±1 kb of a candidate gene. The remaining 175 polymorphisms are associated with a total of 161 candidate genes (Supplementary Table 6). A total of 100 of these polymorphisms are intronic, 29 are exonic with 6 producing nonsynonymous changes to the peptide and 1 a start gain, 10 are located in the 5′ or 3′ untranslated regions, and 36 are within 1 kb up or downstream of the candidate gene (Supplementary Table 5). Of note in this analysis is that the results were not filtered for allele frequency > 0.05. This was a concerted choice; several of the most interesting candidates, including *CG5888* and *ilp5*, would otherwise have been left out of the analysis. The number of total variants analyzed drops from 237 to 90, and the number of candidate genes associated at *P* < 10^−4^ drops from 161 to 75. While this, along with the use of a low stringency *P*-value cut-off, increases the probability of false positives, it likewise increases our power when performing pathway enrichment. Validation of candidate genes with low minor allele frequencies in later studies will distinguish true positives from false positives.

One concern with using an RNAi model to reduce *Sirt1* expression is that the modifiers identified might be specific RNAi efficacy, rather than hyperglycemia. The modifiers could be altering the degree and efficiency of *Sirt1* knockdown, so that hyperglycemia is actually correlating with the amount of *Sirt1* expression that is achieved. If this was the case, we would expect to see: 1—correlation of wild-type *Sirt1* expression with glucose levels across the DGRP: 2—the top candidate modifiers associated with RNAi machinery or the efficiency of the *GAL4/UAS* system: and 3—differences in *Sirt1* expression between *r4>Sirt1-RNAi/DGRP* lines determined to have low versus high glucose measurements. None of these appear to be the case. We do not see candidate genes with functions in RNAi, either from a single-gene function perspective or when looking at enriched gene categories (Supplementary Tables 6 and 7). We do not see any correlation in *Sirt1* expression with glucose levels in corresponding DGRP lines (Supplementary Fig. 7a). We also see no significant difference in *Sirt1* expression between experimental lines associated with high or low glucose, as determined by qPCR (Supplementary Fig. 7b). Furthermore, the *GAL4/UAS* system is commonly used to model disease in the DGRP, and the candidates identified have always been unique to the disease and, at times, even the specific model in question (He *et al.* 2014; Chow *et al.* 2016; Lavoy *et al.* 2018; Palu *et al.* 2019; Talsness *et al.* 2020). All of this suggests that the candidate genes identified through this screen are modifying *Sirt1*-associated hyperglycemia directly rather than altering the degree of *Sirt1* knockdown.

### Candidate modifiers of *Sirt1* are involved in basic metabolic processes, the immune response, and the regulation of neuronal communication

Because loss of *Sirt1* in the fat body alters glucose metabolism and insulin sensitivity in the organism, we expected modifiers of hyperglycemia to impact pathways linked to central carbon metabolism as well as external processes that influence secretion and signaling of hormones such as insulin. To determine if this is the case, we examined the individual functions of the top GWA candidates and looked for pathways and processes that are enriched in this list. While we attempted first to do this through Gene Ontology analysis of our top candidates, we found no significantly enriched terms. We therefore utilized individual known physical interactions and GO term enrichment through GSEA to highlight likely candidate pathways.

#### Analysis of candidate modifiers

We expected our top candidates to include genes that function in pathways or processes related to *Sirt1* regulation or activity. Among the most interesting candidates are those involved in NAD metabolism, as NAD is an important cofactor in the *Sirt1* enzymatic reaction (Nogueiras *et al.* 2012). *NDUFS4* and *ND-PDSW* both encode parts of the NADH dehydrogenase component of Complex I in the electron transport chain (FlyBase Curators 2008; Gaudet *et al.* 2011). There are also 2 NADP kinases, enzymes involved in the generation of NADP from NAD (*CG33156* and *CG6145*) (Gaudet *et al.* 2011). *DUOX*, an NADPH oxidase, passes electrons from NADPH to oxygen, generating hydrogen peroxide and altering the redox balance of the mitochondria and, by extension, the cell (Anh *et al.* 2011; Gaudet *et al.* 2011; Willems *et al.* 2015).

Genes involved in central glucose metabolism as well as insulin signaling are also candidate modifiers. MFS5 acts as a transporter of both glucose and trehalose for the uptake of these sugars from circulation (McMullen *et al.* 2021). Glucose-6-phosphatase (*G6P*) is the last rate-limiting step in both gluconeogenesis and glycogenolysis, which are used to generate glucose for release into the body during fasting (Gaudet *et al.* 2011; Lizák *et al.* 2019). These 2 genes directly regulate circulating glucose levels. Candidates involved in other metabolic pathways include *CTPSyn*, which encodes the rate limiting step in cytidine synthesis, the very long chain fatty acid ligase *bgm*, the mannosidase *Edem2*, and the oxoglutarate dehydrogenase complex subunit *CG33791* (Kang and Ryoo 2009; Gaudet *et al.* 2011; Jang *et al.* 2015; Sivachenko *et al.* 2016; Zhou *et al.* 2019).

Partially responsible for regulating general metabolic flux through these various pathways is insulin. Interestingly, a top candidate is *ilp5*, one of several insulin-like peptides expressed in the insulin-producing cells (IPCs) in the *Drosophila* brain (Géminard *et al.* 2009). Our analysis also identified *IA-2*, a phosphatase involved in ilp secretion, *CG4168*, an uncharacterized gene whose closest human ortholog *IGFALS* encodes a protein that binds to and stabilizes IGF proteins in circulation, and *wrd*, a subunit in the protein phosphatase PP2A that negatively regulates insulin and TOR signaling (Boisclair *et al.* 1996; Kim *et al.* 2008; Hahn *et al.* 2010). Other potential modifiers of insulin stability and signaling in circulation are *dally*, *cow*, and *Hs3st-A*. Both *dally* and *cow* encode heparin sulfate proteoglycans, while *Hs3st-A* encodes an O-sulfotransferase that acts on these proteoglycans (Filmus and Selleck 2001; Gaudet *et al.* 2011; Chang and Sun 2014). Previous work has demonstrated an impact of the enzyme heparanase, which cleaves heparan sulfate, on diabetic autoimmunity and complications such as nephropathy (Rabelink *et al.* 2017). While this is more peripheral to the central insulin signaling pathway in *Drosophila*, it highlights the utility of such factors in altering disease processes in subtle ways.

Another interesting group of candidates are those associated with neuronal development and function. Several members of the defective proboscis extension response (*dpr*) family were represented in our list (*dpr2*, *dpr6*, and *dpr13*) along with the *dpr*-interacting protein *DIP-eta*. The *dpr* gene family is collectively associated with synapse organization and function, as are the candidate genes *fife*, *CG32373*, and *atilla* (FlyBase Curators *et al.* 2004; Kurusu *et al.* 2008; Carrillo *et al.* 2015; Bruckner *et al.* 2017). We also noted candidates involved in neuropeptide signaling (*rk* and *RYa-R*), voltage-gated potassium channels and their regulation (*CG5888* and *CG1688*), and axon guidance (*tutl*, *CadN2*, *CG34353*, and *sbb*) (Rao *et al.* 2000; Luo *et al.* 2005; Prakash *et al.* 2005; Al-Anzi and Wyman 2009; Gaudet *et al.* 2011; Ida *et al.* 2011). The IPCs in *Drosophila* are actually neuroendocrine cells located in the brain, as are the AKH-producing cells responsible for secreting the glucagon-like hormone AKH (Géminard *et al.* 2006). The secretion of insulin is therefore dependent upon the correct development, connection, and signaling of neuronal cells.

The immune response is another generally enriched category of modifier genes. Several members of the nimrod family of immunoglobulins (*NimB2*, *NimC1*, and *NimC3*) were identified by GWA. All are implicated in the innate immune response, with *NimC1* and *NimC3* in particular having roles in phagocytosis (Somogyi *et al.* 2010). In response to insecticides, *LRR* regulates the immune response through NF-kappaB, whose activation is an early protective event in the progression and pathology of diabetes (Di Prisco *et al.* 2013; Irvin *et al.* 2018). Two lysozyme enzymes with links to bacterial defense (*LysX* and *CG7798*) highlight the role of oxidative stress and redox homeostasis in the innate immune response (FlyBase Curators 2008). As Sirt1 has roles in regulating the response to oxidative stress, we looked for other genes with similar functions (Brunet *et al.* 2004). Besides *DUOX* (Anh *et al.* 2011), *CG42331* encodes a peroxidase that appears to be strongly enriched in the pupal fat body, and *cyp28a5* encodes an oxidoreductase that, similar to *LRR*, is involved in the response to insecticides (FlyBase Curators *et al.* 2004; Gaudet *et al.* 2011; Graveley *et al.* 2011). It is now believed that Type I and Type II diabetics both suffer at least to some degree from autoimmunity (de Candia *et al.* 2019). Exploring the direct and indirect connections of *Sirt1* to the immune response and oxidative stress directly is an interesting avenue for future direction.

#### Physical interaction network

**Fig. 3.**
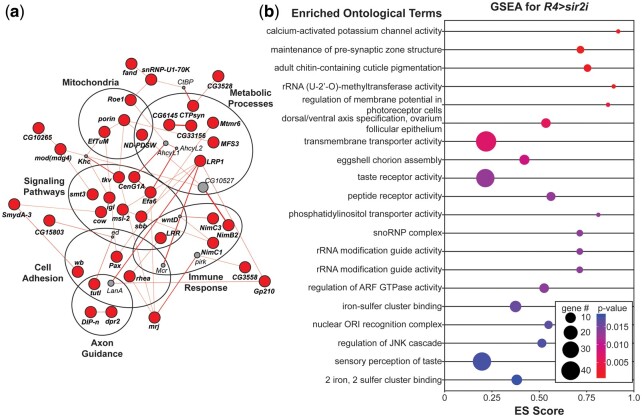
Immune responses, neuronal function, and basic metabolic processes are overrepresented in GWA candidate modifiers of hyperglycemia. a) *r4>Sirt1-RNAi* modifier network, as plotted by the GeneMANIA plugin in Cytoscape (Shannon *et al.* 2003; Montojo *et al.* 2010). Significant candidate modifiers are indicated in bold and darkened, with physical interactions indicated by connecting lines. Thicker lines indicate stronger evidence for the interaction. Encircled genes share common pathways or functions. Interacting genes outside of the candidate modifier list are indicated by lighter circles. b) Top 20 significant ontological categories as identified by GSEA. Categories are arranged from most significant on top to least significant along the *y*-axis. *P*-values are indicated by a gradient, with red the lowest *P*-values and blue the highest *P*-values. Enrichment score (ES) for each category is plotted along the *x*-axis. Gene number identified in each category is indicated by dot size.

We generated a network of physical interactions among the 161 candidate genes identified above. These were identified and visualized using Cytoscape software with the GeneMania plugin (Shannon *et al.* 2003; Montojo *et al.* 2010). The products of 37/161 candidate genes were found to physically interact with at least one other candidate gene product with no more than one bridging node represented by a noncandidate gene (Fig. 3a). This high degree of interaction suggests that the modifiers identified in this screen are indeed functioning through shared processes.

Focusing then on the 37 genes involved in physical interactions, we identified several broad functional categories that could influence glucose homeostasis in the fly. The most obvious category are enzymes that catalyze steps in basic metabolic pathways (*N* = 7). This includes NAD kinases (*CG6145* and *CG33156*) and NADH dehydrogenase (*ND-PDSW*) (Gaudet *et al.* 2011). Other metabolic candidate modifiers include *MFS3* and *CTPsyn* (Zhou *et al.* 2019). *Mtmr6* encodes a phosphatidylinositol phosphatase, a key enzyme in insulin signaling (Gaudet *et al.* 2011). Also interesting is the gene *LRP1*, which is orthologous to human LDL receptor related protein 1. In additional to its role in lipid homeostasis, *LRP1* has also been implicated in Alzheimer’s disease, for which metabolic disease and obesity are risk factors (Kang *et al.* 2000; Anstey *et al.* 2011).

Curiously, several of the genes highlighted in this analysis also happen to localize specifically to the mitochondria (*N* = 4). *Roe1* and *porin* are both transporters involved in the import of molecules into the mitochondria (Komarov *et al.* 2004; Jana Alonso *et al.* 2005; FlyBase Curators 2008; Gaudet *et al.* 2011). The NADH dehydrogenases *ND-PDSW* and *NDUFS3* both function in the mitochondria as well as part of Complex I in the electron transport chain (Jana Alonso *et al.* 2005). Closer examination of the top GWA candidates reveals additional mitochondrial localization candidates including the amino acyl tRNA synthetase *GlyRS*, the membrane bound regulator of protein kinase A (*pkaap*) and the translation elongation factor *mEFTu1* (Gaudet *et al.* 2011; Lu *et al.* 2015). In adult metabolic homeostasis, central carbon metabolism is generally used to fuel the electron transport chain in the mitochondria and generate ATP for the cell (Barry and Thummel 2016). Altering the activity of this essential downstream pathway could have a clear impact on glucose utilization and disease progression in diabetes.

Similar to our examination of top candidates, our physical interaction map highlighted the immune response (*NimC1*, *NimC3*, *NimB2*, and *LRR*) and neuronal function. *Tutl*, *dpr2*, and *DIP-eta*, and *wb* are all involved in synapse organization and axon guidance, while *Pax* and *rhea* are involved in focal adhesion (Delon and Brown 2009). The identification of genes important for cellular communication suggests that some of the modifiers identified in this study have roles in tissues other than the fat body, such as the IPC and APC neurons. This is an important avenue of future exploration.

#### Gene set enrichment analysis

In the second approach, we performed gene set enrichment analysis (GSEA) analysis to identify gene ontology terms for which associated variants are enriched. Unlike traditional GO analysis, which relies upon a set of genes based on a *P*-value cut-off, GSEA examines the entire gene set (Dyer *et al.* 2008). For each defined GO category, GSEA determines whether the members of that category are randomly distributed throughout the ranked gene list provided or if they are enriched for the lower *P*-values found at the top of that list. GO categories enriched at the top of the list describe important functions of the gene set. GSEA identified 52 significantly associated gene sets (≥3 genes) with positive enrichment scores at a *P*-value of <0.05 (Supplementary Table 7, Fig. 3b). The top 2 gene sets implicate neuronal function and communication in the *Sirt1i*-associated hyperglycemia phenotype: calcium-activated potassium channel activity (GO: 0015269, *P* = 1.1E-3) and maintenance of presynaptic active zone structure (GO: 0048790, *P* = 1.2E-3). Similar categories can be found through the list of significantly associated gene sets, including dendrite morphogenesis (GO: 0048813, *P* = 0.049), which represents the largest group of genes at *N* = 119 and contains 2 of the top GWA candidates (*slit* and *fruitless*). Coupled with the neuronal genes identified in our physical interaction network, this suggests that function in the neuroendocrine cells could play a big role in glucose homeostasis in the fat body.

Also enriched are taste receptor activity (GO: 0008527, *P* = 0.013) and sensory reception of taste (GO: 0050909, *P* = 0.018). These categories highlight feeding and diet as a possible source of variation in glucose. As expected, we also see evidence of general metabolic processes. Some, like alpha, alpha-trehalase activity (GO:0004555, *P* = 0.043) and phosphatidylinositol transporter activity (GO:0008526, *P* = 0.013) have direct links to glucose metabolism and insulin signaling. Others, such as oxysterol binding (GO:0008142, *P* = 0.026), glutamate biosynthetic process (GO:0006537, *P* = 0.043), and isoprenoid biosynthetic process (GO:0008299, *P* = 0.048) function more peripherally to carbon metabolism and are likely influencing hyperglycemia by their general contribution to physiological homeostasis.

Another interesting group of GO categories highlighted by GSEA are RNA processing functions. rRNA (uridine-2′-O-)-methyltransferase activity (GO:0008650, *P* = 2.4E-3) is the fourth most associated category as ranked by *P*-value, and others such as snoRNA binding (GO:0030515, *P* = 0.014) reiterating this function. The presence of RNA processing categories is of particular interest because 3 of the top candidate genes by GWA are splicing factors (*bru1*, *fand*, and *snRNP-U1-70k*) (Park *et al.* 2004; Oas *et al.* 2014; Spletter *et al.* 2015). While it is unclear how rRNA or mRNA processing may directly or indirectly influence glucose homeostasis in particular, the identification of this process through several different methods of analysis is striking and worth further exploration.

### Functional analysis of candidate modifier genes

**Fig. 4.**
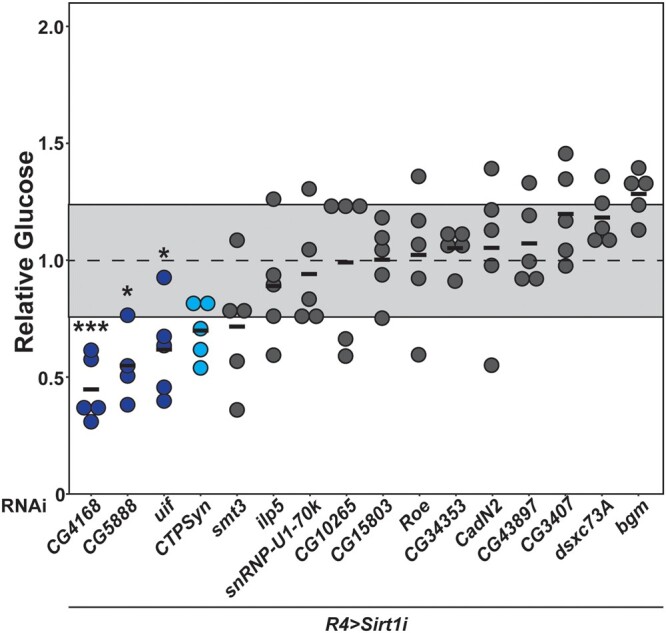
Loss of candidate gene expression suppresses hyperglycemia in the *r4>Sirt1-RNAi* model. RNAi against candidate modifiers was expressed under the control of *r4-GAL4* in the *r4>Sirt1-RNAi* model. Glucose level for each sample was normalized to the levels in a genetically matched control line crossed into the *R4-Sirt1* line. Average *r4>Sirt1-RNAi* control glucose levels after normalization are indicated by a dotted line at 1.0, with standard deviation highlighted by the gray box. Whole fly glucose concentration was quantified for *N* = 4–5 samples per strain, each consisting of 5 flies and individually plotted along the *y*-axis. Knockdown of *CG4168, CG5888*, or *uif* significantly reduces glucose concentrations in the *r4>Sirt1-RNAi* model of hyperglycemia compared to controls (blue). Loss of *CTPSyn* does not significantly alter glucose levels, but a trending decrease in glucose levels were observed in several independent RNAi strains (light blue, data not shown). Loss of *smt3*, *ilp5*, *snRNP-U1-70k*, *CG10265*, *CG15803*, *Roe*, *CG34353*, *CadN2*, *CG43897*, *CG3407*, *dsxc73A*, or *bgm* do not produce a significant effect (dark gray). *P*-values were calculated using 1-way ANOVA followed by Dunnett’s multiple testing correction. *P < 0.05, ***P < 0.001.

To confirm the roles of our candidate genes in regulating glucose homeostasis, we elected to test the impact of loss of modifier expression for 16 of the most significant candidates for which we were able to obtain transgenic RNAi lines (Table 1). We crossed the RNAi strains targeting each of these modifiers into the *r4>Sirt1-RNAi* line, aged the resulting progeny for 2–3 weeks, and measured glucose in fasted males. We also measured protein levels as a control. Knockdown of modifier genes did not significantly alter protein levels as compared to a genetically matched control (Supplementary Fig. 8). Knockdown of the genes *CG4168*, *CG5888*, and *uif* resulted in suppression of the hyperglycemia phenotype, with a significant decrease in glucose content per fly compared to controls expressing *r4>Sirt1-RNAi* (Fig. 4). Loss of the other candidates did not significantly impact glucose content compared to controls (Fig. 4). All 3 candidate suppressors are predicted to be expressed at low levels in the fat body (Supplementary Table 8), although the expression of these genes as previously measured in wild-type males does not correlate with glucose levels in the corresponding *r4>Sirt1-RNAi/DGRP* strains (Supplementary Fig. 9, a–c). However, reduced expression for *CG4168* and *uif* was confirmed in the *r4>Sirt1-RNAi* background for the respective RNAi strains. Ultimately, this confirmation of reduced expression combined with the phenotypic change supports the hypothesis of *CG4168* and *uif* as modifier genes (Supplementary Fig. 10, a and b). We were unable to confirm reduced expression for *CG5888* (Supplementary Fig. 10c).

**Table 1.** Top tested candidate modifiers of R4>sir2i associated hyperglycemia.

*P*-score	FlyBaseID	Gene name	Human ortholog(s)	Function
2.80E-07	FBgn0028523	*CG5888*	*LRRC26* and *LRRC52*	Voltage-gated potassium channel
1.43E-06	FBgn0261799	*dsx-c73A*	—	Ribosome-associated, elongation factor GTPase
2.09E-06	FBgn0031879	*Uif*	*ELAPOR1*	Notch signaling
2.35E-06	FBgn0262018	*CadN2*	Multiple Cadherin genes	Cell adhesion, axon guidance
2.64E-06	FBgn0033990	*CG10265*	—	Unknown
3.06E-06	FBgn0027348	*Bgm*	*ACSBG1* and *ACSBG2*	Long-chain fatty acid ligase
6.09E-06	FBgn0028888	*CG4168*	*IGFALS*	Serum IGF-binding protein
6.25E-06	FBgn0016978	*snRNP-U1-70K*	*SNRNP70*	Spliceosome component
6.25E-06	FBgn0264922	*smt3*	*SUMO1* through *4*	ONLY Drosophila SUMO protein
6.80E-06	FBgn0038606	*CG15803*	*MPDZ*	C-terminal binding protein
7.09E-06	FBgn0031573	*CG3407*	Various ZFN transcription factors	Zinc finger nuclease transcription factor
7.41E-06	FBgn0085382	*CG34353*	*IGLON5*, *LSAMP*, and *NEGR1*	Cell adhesion, axon guidance
7.94E-06	FBgn0014877	*Roe1*	*GRPEL1*	Mitochondrial PAM complex
8.29E-06	FBgn0264489	*CG43897*	*BRD4*, *LDB3*, *PDLIM1*, etc	Actin/cytoskeletal organization
8.29E-06	FBgn0044048	*Ilp5*	*INS*, *INS-IGF2*	Insulin signaling
9.21E-06	FBgn0266452	*CTPsyn*	*CTPS1* and *CTPS2*	CTP synthase

Candidate modifier genes identified via GWA (*P* < 1E-5), RNAi available.

Considering that a top GSEA category include genes involved in RNA processing functions, it is possible that the change in phenotype upon reduction in expression of modifier genes might be due to changes in *Sirt1* RNAi efficiency or *Sirt1* expression. To rule this out, we examined *Sirt1* expression upon expression of each candidate suppressor RNAi construct in the *r4>Sirt1-RNAi* background. *Sirt1* expression was not increased, or indeed significantly changed, upon reduced expression of any of the candidate suppressor genes (Supplementary Fig. 10d). This indicates that the improved phenotype is probably not due to a change in the efficiency of *Sirt1* RNAi. These results demonstrate that a subset of the top GWA candidate modifiers are capable of modifying the hyperglycemia phenotypes associated with the *r4>Sirt1-RNAi* model of diabetes.

## Discussion

Identifying and characterizing the genetic factors influencing the severity of diabetes is critical to early diagnosis. Prevention is still the best strategy available, and providing patients at high risk for complications with knowledge of that risk could prevent the worst symptoms from manifesting. It could even enable intervention before the progression of disease is irreversible.

In this study, we identified and analyzed a number of candidate modifiers of hyperglycemia in a previously characterized model of diabetes, *Sirt1* loss of function. We used the DGRP as an unbiased source of natural genetic variation for this screen. This is the first time a genetic model of metabolic dysfunction has been put to this use, as previous screens have either focused on dietary stress as a source of metabolic disease or on the impact of genotype on metabolic parameters under nonstressed conditions (Mackay *et al.* 2012; Ivanov *et al.* 2015; Nelson *et al.* 2016; Jehrke *et al.* 2018; Everman *et al.* 2019). We observed very little overlap in modifier candidates between our observations and these studies. This is consistent with previous work demonstrating that even when the observed phenotypes are similar or nearly identical, the overlap in modifiers is often small in different models of disease (Palu *et al.* 2019). One exception is a screen for the response to starvation resistance performed by Everman *et al.* in 2018. They identified *CG15803*, a transporter of unknown function that was also identified in our studies (Everman and Morgan 2018). While our preliminary analysis suggests that expression of this gene is not required in the fat body, it is also possible that expression of *CG15803* was not significantly or sufficiently reduced by expression of the RNAi construct to influence the phenotype. Further validation of the efficiency of the RNAi in this study is necessary to confirm this conclusion. Alternatively, *CG15803* could have a role in another physiologically relevant tissue such as the IPCs or APCs. Indeed, *CG15803* appears to be most highly expressed in the head and CNS (Graveley *et al.* 2011). This is an interesting avenue of future exploration.

We did observe overlap in general gene categories with previous studies, even when direct overlaps were few. This was found to be true for modifiers of neuronal function. In a broad exploration of genetic variation in the nutrient response that looked at triglycerides, starvation resistance, mass, and glucose, *NimB3* was identified as a candidate modifier (Nelson *et al.* 2016). While *NimB3* was not identified in this screen, we did find *NimB2*, *NimC1*, and *NimC3*. *Fife*, which also has roles in synapse organization, was identified in this study and one for starvation resistance (Mackay *et al.* 2012). As mentioned above, both the IPCs and APCs are neuroendocrine cells found in or near the brain (Géminard *et al.* 2006). Maintenance of neuronal function would be critical to hormonal balance as a result. Furthermore, it is broadly acknowledged that metabolic homeostasis is also dependent upon feeding rate, over which the central nervous system has some sway (Owusu-Ansah and Perrimon 2014). The identification of neuronal genes in each analysis suggests that regulation of particular neuronal pathways and cells is critical to the maintenance of physiological homeostasis.

Given the prevalence of neuronal and sensory perception genes in the analysis, a concern could be raised for the role feeding rate could be playing in the variation of glucose across the DGRP. While all flies were collected under identical conditions and were maintained on the same diet, it is nonetheless possible that some may simply be eating more due to differences in the sensing of satiation or to differences in perception of taste. These differences in perception and consumption can have detectable impacts on metabolic phenotypes (May *et al.* 2019). To assess this, we compared glucose with male feeding rate in a previous analysis (Supplementary Table 4) (Garlapow *et al.* 2015). We saw no correlation whatsoever, indicating that while nutrient sensing may play a role in the response to *r4>Sirt1-RNAi-*induced hyperglycemia, it is not the driving factor in the variation observed in this screen (Supplementary Fig. 11).

As Sirt1 utilizes NAD as a cofactor during its enzymatic reaction, altering the balance of NAD in the cell through differential regulation of these enzymes could further impact the activity of other pathways that require NAD as an electron carrier, or exacerbate the phenotypes associated with *Sirt1* loss-of function (Nogueiras *et al.* 2012). It is therefore interesting that we identified enzymes involved in NAD(P) metabolism as candidate modifiers, along with other genes related to the regulation of redox homeostasis and oxidative stress. While these are primarily mitochondrial enzymes rather than cytosolic enzymes, it is well-established that disruption of NAD+/NADH ratios in the various cellular compartments can influence the same in other compartments (Cantó *et al.* 2015). The same can be said for general redox homeostasis (Willems *et al.* 2015). Many of these enzymes are also critical portions of the innate immune response. While it has long been known that Type I diabetes is an autoimmune disorder, it has recently been acknowledged that Type II diabetics also display symptoms of autoimmunity (de Candia *et al.* 2019). Furthermore, insulin resistance has frequently been associated with inflammation, and the presence of macrophages in the adipose tissue is a hallmark of obesity and diabetes (Wu and Ballantyne 2020). Modifiers associated with innate immunity serve therefore as validation to the study as a whole, and examination of these genes and their function in the context of the *Sirt1* loss-of-function model will be an intriguing focus of future research.

An important component of this study is the validation of top candidate modifiers using RNAi-mediated knockdown of gene expression. We obtained strains expression transgenic RNAi constructs targeting 16 of the most significant candidates (Table 1). We found that constructs targeting *CG4168*, *CG5888*, and *uif* specifically in the fat body resulted in significant suppression of hyperglycemia (Fig. 4). These 3 genes are therefore our top candidate suppressor modifiers. While expression of these genes in wild-type adult DGRP males does not correlate with glucose levels in the corresponding *r4>Sirt1-RNAi*/DGRP strains (Supplementary Fig. 9) (Huang *et al.* 2015), we were able to confirm reduction in expression for 2 of these genes (*CG4168* and *uif*), supporting them as suppressor modifiers with functions in the fat body (Supplementary Fig. 10, a and b. The lack of correlation can be explained by the change in DGRP genotype (wild-type vs *r4>Sirt1-RNAi*) and tissue of interest (whole fly vs fat body). A transcriptional study more specifically focused on the disease model would likely produce more associated candidate modifiers, and could be a fascinating direction for future exploration.

We also noted a consistent, though not significant, decrease in glucose for 2 independent RNAi constructs targeting *CTPSyn*, suggesting that this gene warrants further study (Fig. 4, data not shown). It was also highlighted in the physical interaction network (Fig. 3). *CTPSyn* functions in pathways critically dependent upon central carbon metabolism, and its inclusion supports a role for secondary metabolic pathways as a sink for increased circulating glucose. The remainder of the genes had no significant or consistent impact on hyperglycemia in the model of *Sirt1* loss of function (Fig. 4). None of the tested modifiers acted as enhancers of the phenotype: loss of modifier expression did not lead to increased glucose levels for any of the tested genes. This could be because hyperglycemia is already quite strong in the *Sirt1* loss-of-function model, or because we simply did not hit on any enhancing modifiers. Of greater concern is the lack of any response for 13 of the 16 tested candidates. One explanation may be found in the large number of known neuronal genes identified in this analysis. This modifier RNAi screen specifically focused on the expression of the RNAi against the candidate genes in the fat body, where expression of *Sirt1* is also reduced. If, however, the function of a modifier gene is primarily concentrated in the IPCs, as with *ilp5*, reducing its expression in the fat body would have little effect on the disease phenotypes in question. We will examine the role of modifier genes not only in the fat body but in the IPCs, APCs, and other physiologically relevant tissues in future work.

Of immediate interest is of course the mechanism of action for the 2 suppressor genes that were confirmed by RNAi in the fat body. The third suppressor, *CG5888*, could not be confirmed as we were unable to detect a change in expression of this gene in the fat body upon expression of the corresponding RNAi construct. Further work will therefore be required to determine if *CG5888*, the top GWA candidate (*P* = 2.80E-07), is capable of modifying hyperglycemia in the *r4>Sirt1-RNAi* model, and if so, in which tissues it is acting.

*Uninflatable* (*uif*) encodes a single pass transmembrane protein found on the apical membrane of epithelial cells and has been found to enable Notch signaling (Loubéry *et al.* 2014). It has also been found to exacerbate disease in a *Drosophila* model of muscular dystrophy (Kucherenko *et al.* 2011), and its closest human ortholog *ELAPOR1* is a regulator of apoptosis and autophagy (Deng *et al.* 2010). Both of these processes are commonly disrupted through inappropriate activation in metabolic disease, and might provide some explanation for the impact of *uif* on *Sirt1i*-associated phenotypes (Bugliani *et al.* 2019).

Perhaps the most intriguing finding is *CG4168* as the modifier with the strongest impact on *Sirt1i*-associated hyperglycemia. The protein encoded by *CG4168* is of unknown function, but its closest human ortholog (*IGFALS*) encodes a serum protein that binds to insulin-like growth factors (IGF) in circulation (Boisclair *et al.* 1996). In mammals, association with IGFALS increases the half-life of insulin-like growth factors in the serum as well as their retention in circulation. While studies of IGFALS in mammals has not shown a role for it in regulating insulin signaling, the *Drosophila* ilp peptides are used for both IGF and insulin signaling activation (Géminard *et al.* 2006). It is possible that secretion of the factor encoded by *CG4168* from the fat body could increase ilp retention in circulation, whereas its loss could result in faster clearance of ilps from circulation. Under conditions that promote insulin resistance, such as the loss of *Sirt1*, it is possible that reduced ilp levels in the hemolymph could slow or prevent insulin resistance and hyperglycemia. Further exploration of the mechanisms behind the action of *CG4168* could reveal important insights into circulating insulin-binding factors and their role in diabetes.

In conclusion, we have identified a number of pathways and processes involved in the degree of hyperglycemia in a genetic model of diabetes. Examination of the candidate genes and pathways described above in this model as well as other models of metabolic dysfunction will shed new light on the mechanism by which insulin resistance and related complications disease onset, progression, and severity. Furthermore, the candidates identified as suppressors could serve as promising targets for therapeutics in diabetes and related metabolic disorders.

## Data availability

Strains and stocks are available upon request, as is code for GSEA. Genomic sequence for the DGRP is available at http://dgrp.gnets.ncsu.edu/. Supplemental material is available at FigShare (https://doi.org/10.6084/m9.figshare.16587326).
